# Epidemiological profile of surgical treatment of lung cancer: retrospective analysis, Brazil, 2014-2023

**DOI:** 10.1590/S2237-96222025v34e20240427.en

**Published:** 2025-08-04

**Authors:** Arthur Minas Alberti, Lucas Kieling, Pedro Bortoluzzi Escobar da Silva, Fernando Silvestre Azambuja, Luísa Godoy, Franco Piccolotto Concolatto, Gabriele Eckerdt Lech, Airton Tetelbom Stein

**Affiliations:** 1 Universidade Federal de Ciências da Saúde de Porto Alegre, Faculdade de Medicina, Porto Alegre, RS, Brazil; 2 Pontíficia Universidade Católica do Rio Grande do Sul, Faculdade de Medicina, Porto Alegre, RS, Brazil

**Keywords:** Lung Neoplasms, Operative Surgical Procedures, Health Inequalities, Socioeconomic Factors, Retrospective Studies, Neoplasias Pulmonares, Procedimientos Quirúrgicos Operatorios, Desigualdades en Salud, Factores Socioeconómicos, Estudios Retrospectivos

## Abstract

**Objective:**

To analyze the socioeconomic influences on the surgical treatment of lung cancer in Brazil and to provide reflection points to propose more effective strategies for tackling this disease.

**Methods:**

Data from 2014 to 2023 were extracted from the Hospital Information System of the Brazilian Unified Health System and the Brazilian Institute of Geography and Statistics. Sex and region of origin of patients who underwent surgical procedures for lung cancer were analyzed. Statistical analyses were performed using GraphPadPrism software version 8.0.1 and R software version 4.4.2.

**Results:**

20,805 surgical procedures and 112,118 diagnoses of malignant neoplasms of the bronchi and lungs in Brazil were analyzed between 2014 and 2023. Women accounted for 50.6% of the procedures. Men accounted for 54.7% of the diagnoses. There was no significant difference between sexes in terms of procedures performed or consultations (p-value>0.05). The South region had the highest rate of procedures (20.1/1 million inhabitants/year). The North region had the lowest rate of procedures (3.5/1 million). A 51.6% increase in procedures was identified over the decade, with positive correlations between the Human Development Index (r=0.62; p-value<0.001) and per capita income (r=0.48; p-value 0.010) with procedures.

**Conclusion:**

Socioeconomic factors appear to influence access to surgical treatment of lung cancer in Brazil, with significant regional disparities, especially in the North and Northeast regions. The increase in surgical procedures reflects technological advances and public policies, but inequalities in access persist. Investments in infrastructure and equitable policies are essential to address these inequalities.

## Resumen

**Objetivo:**

Analizar las influencias socioeconómicas en el tratamiento quirúrgico del cáncer de pulmón en Brasil y proporcionar reflexiones para proponer estrategias más efectivas para el enfrentamiento de esta enfermedad.

**Métodos:**

Los datos de 2014 a 2023 fueron extraídos del Sistema de Información Hospitalaria del Sistema Único de Salud de Brasil y del Instituto Brasileño de Geografía y Estadística. Se analizó el sexo y la región de procedencia de los pacientes sometidos a procedimientos quirúrgicos por cáncer de pulmón. Los análisis estadísticos se realizaron utilizando el software GraphPadPrism 8.0.1 y R (versión 4.4.2).

**Resultados:**

Se analizaron 20.805 procedimientos quirúrgicos y 112.118 diagnósticos de neoplasias malignas de bronquios y pulmones en Brasil entre 2014 y 2023. Las mujeres representaron el 50,6% de los procedimientos. Los hombres representaron el 54,7% de los diagnósticos. No hubo diferencia significativa entre sexos en la realización de procedimientos o consultas (p-valor>0,05). La región Sur presentó la mayor tasa de procedimientos (20,1/1 millón de habitantes/año). La región Norte tuvo el valor más bajo (3,5/1 millón). Se identificó un incremento de 51,6% en los procedimientos a lo largo de la década, con correlaciones positivas entre el índice de desarrollo humano (r=0,62; p-valor<0,001) y el ingreso per cápita (r=0,48; p-valor 0,010) con los procedimientos.

**Conclusión:**

Los factores socioeconómicos parecen influir en el acceso al tratamiento quirúrgico del cáncer de pulmón en Brasil, con disparidades regionales significativas, especialmente en las regiones Norte y Nordeste. El aumento de trámites refleja avances tecnológicos y políticas públicas, pero persisten desigualdades en el acceso. Las inversiones en infraestructura y políticas equitativas son esenciales para abordar estas desigualdades.

**Ethical aspects**

This research used public domain data and anonymized databases.

## Introduction

Lung cancer incidence and mortality present significant public health challenges, with far-reaching implications for health systems and affected communities. Regional disparities in lung cancer mortality in Paraná, Brazil were highlighted, offering insight into the influence of socioeconomic factors in this scenario (1). The correlation between mortality rates and indicators such as the Municipal Development Index highlights the connection between the socioeconomic status of patients and access to treatment (2,3). 

There are analyses in the literature carried out on a national scale. Researchers from the state of Rio Grande do Norte revealed a similar panorama (2), indicating a high prevalence of advanced stage lung cancer diagnoses throughout Brazil. A critical association between factors such as population aging, per capita income and regional disparities was suggested. The relationship between these indicators and the incidence of this disease highlighted the urgent need to understand and address disparities in timely access to health services.

In 2017, Chinese researchers (4) indicated that the influence of the socioeconomic context on the incidence and mortality of lung cancer crossed Brazil’s borders, making it a global phenomenon. The increasing incidence among women in countries such as Brazil, Spain and Cyprus, together with rising mortality rates in more developed regions, has highlighted the complexity of the factors shaping this epidemiological landscape (4).

This article aimed to analyze the socioeconomic influences on the surgical treatment of lung cancer in Brazil. The aim was to identify existing disparities and provide insights to propose more effective and equitable strategies for tackling this disease, by examining this complex relationship between social and biological risk factors.

## Methods

### Study design

This is an observational, cross-sectional and retrospective study, which analyzed data from 2014 to 2023. This was conducted based on the Strengthening the Reporting of Observational Studies in Epidemiology and Sex and Gender Equity in Research protocols, aiming to increase the transparency of the study and ensure gender equity.

### Context

Data were collected from two Brazilian secondary databases: the Hospital Information System of the Unified Health System (SIH-SUS) and the Brazilian Institute of Geography and Statistics (IBGE). The information referred to the number of surgical procedures performed throughout the national territory, stratifying it into five geographic macro-regions: North, Northeast, Midwest, Southeast and South. The period analyzed was from January 1, 2014 to December 31, 2023.

### Participants

All patients who underwent surgical procedures for the treatment of lung cancer financed by the SUS (Brazilian Unified Health System), registered in the SIH-SUS, with diagnoses classified under the International Statistical Classification of Diseases and Related Health Problems (ICD-10) C34 (malignant neoplasm of the bronchi and lungs) were included. No exclusion criteria were applied, ensuring the inclusion of all hospitalizations recorded during the study period.

### Variables

The variables analyzed included demographic aspects, such as sex, age group and ethnicity; clinical characteristics, such as the number of diagnoses and surgical procedures performed; and socioeconomic factors, such as the Human Development Index (HDI) and per capita income by geographic macro-region.

### Data sources /measurement

Clinical and demographic data were obtained from SIH-SUS, while socioeconomic data were extracted from the 2022 IBGE Demographic Census. The information was standardized for analysis, with the procedure index adjusted to 1 million inhabitants per state and region. 

### Bias

Efforts were made to minimize biases, including the use of official and widely validated sources, such as SIH-SUS and IBGE, and the implementation of population adjustments to compare procedure rates between states and regions. An assessment of the normality of the data was carried out prior to the statistical analyses, ensuring the validity of the tests applied.

### Study size

The sample size was determined by the totality of data available during the study period. A total of 20,805 surgical procedures performed and 112,118 diagnoses of malignant neoplasms of the bronchi and lungs recorded between 2014 and 2023 were analyzed.

### Statistical methods

Descriptive analyses according to sex and macro-region were reported with frequencies and percentages. For categorical variables, the following were performed: Shapiro-Wilk tests to verify data normality; unpaired t-test to compare genders; Prais-Winsten model to analyze annual trends in the number of diagnoses and procedures; one-way analysis of variance test with post hoc Tukey’s correlation to assess regional differences; and Spearman’s correlation to identify associations between HDI, per capita income and number of procedures. Statistical analyses were performed using GraphPad Prism software v.8.0.1 and R software v.4.4.2.

## Results

Between 2014 and 2023, 20,805 pulmonary surgical procedures were reported, as well as 112,118 diagnoses of malignant neoplasms of the bronchi and lungs, according to ICD-10 C34. Of the total number of surgical procedures performed, 10,529 (50.6%) were in women and 10,276 (49.4%) in men. Regarding lung cancer diagnoses, 61,273 (54.7%) were in men and 50,845 (45.4%) in women.

**Table 1:** Comparison of mean differences in adjusted procedure indices with Tukey test, 95% confidence intervals (95%CI) and adjusted p-values. Brazilian regions, 2014-2023

Regions	Mean difference	(95%CI) of the difference	p-value
North versus Northeast	-2.21	(-4.20; -0.22)	0.023
North vs. Midwest	-2.60	(-4.59; -0.61)	0.005
North vs. Southeast	-8.29	(-10.28; -6.30)	<0.001
North vs. South	-16.56	(-18.55; -14.57)	<0.001
Northeast vs. Midwest	-0.39	(-2.38; 1.60)	0.981
Northeast vs. Southeast	-6.08	(-8.07; -4.09)	<0.001
Northeast vs. South	-14.35	(-16.34; -12.36)	<0.001
Midwest vs. Southeast	-5.69	(-7.68; -3.70)	<0.001
Midwest vs. South	-13.96	(-15.95; -11.97)	<0.001
Southeast vs. South	-8.27	(-10.26; -6.28)	<0.001

The unpaired t-test comparing procedures between men and women was not statistically significant (p-value 0.693). The test was bilateral with a t-statistic of 0.400 and 18 degrees of freedom. The mean number of procedures was 1,028 for men and 1,053 for women, with a difference of 25.30 (standard error of the mean -63.16; 95% confidence interval [95%CI] -107.4; -158.0; p-value 0.693; r^2^=0.009). The F-test comparing variances resulted in an F-statistic of 3.522 with 9 and 9 degrees of freedom and p-value 0.074, indicating no significant difference in variances. For visits to the doctor, the unpaired t-test also resulted in a non-significant difference (p-value 0.064). This was a two-sided test with a t-statistic of 1.970 and 18 degrees of freedom. The mean difference was -1.043 (standard error of the mean 529.4; 95%CI -2.16; 69.46; r^2^=0.777), suggesting a moderate effect size. The F test comparing the variances resulted in the F statistic of 1.604 with 9 and 9 degrees of freedom and p-value 0.492, indicating no significant difference in the variances. The sample size for both columns A and B was 10. 

**Figure 1:**
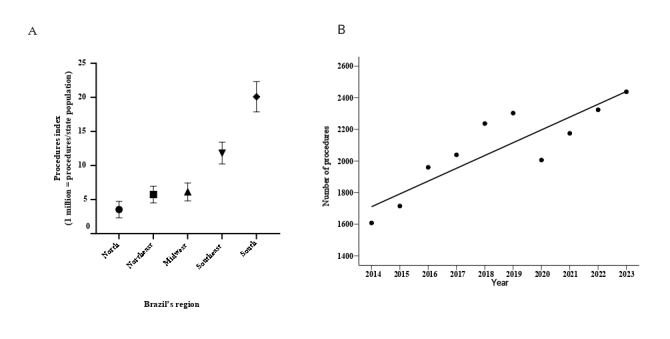
Index of surgical procedures in Brazil’s regions (A) and number of procedures performed in the country from 2014 to 2023 (B)

When comparing the procedure indicators for each region, the South ranked first with 20.1 surgical procedures for resection of lung and bronchial neoplasms per 1 million inhabitants per year. This was followed by the Southeast with 11.8, the Midwest with 6.1, the Northeast with 5.7 and the North with 3.5 surgeries for the treatment of lung cancer per 1 million inhabitants per year (Figure 1A).

A statistically significant upward trend was observed in the number of procedures performed to treat bronchial and pulmonary neoplasms, after correction for serial autocorrelation using the Prais-Winsten model (p-value<0.001) (Figure 1B). By applying this analysis, it was observed that the growing trend in the number of surgical procedures over the years remained significant (coefficient=81.03; 95%CI 43.53; -118.53; p-value<0.001). The Durbin-Watson test indicated a reduction in serial autocorrelation (Durbin-Watson=1.695; p-value 0.045). The difference was 51.6%, with 1,608 procedures in 2014 and 2,438 in 2023 (r^2^=0.876). The analysis of variance test revealed a significant difference between the means of the procedure index of the five regions (F (4,45)=182.2, p-value<0.001). The R^2^ value was 0.942, indicating a good fit of the model, which suggested significant differences between the regional averages. 

**Figure 2:**
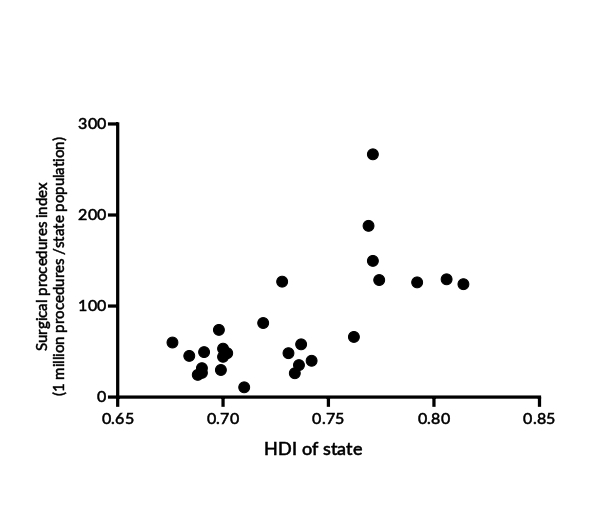
Correlation between the Human Development Index (HDI) of states and their index of surgical procedures. 2014-2023

**Figure 3:**
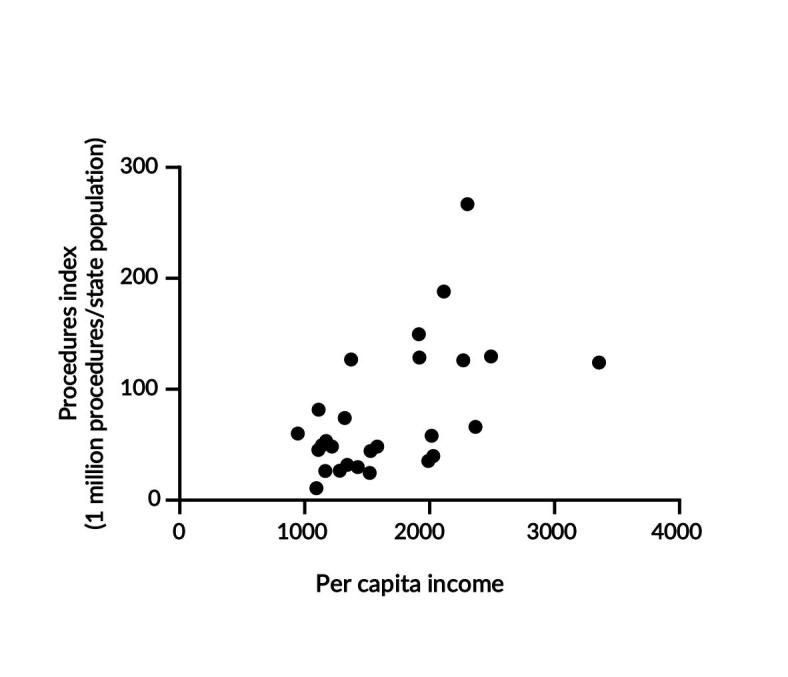
Correlation between *per capita* income in reais (R$) of Brazilian states and their index of surgical procedures. 2014-2023

Positive correlations can be observed between a state’s HDI and the number of surgical procedures, with Spearman’s correlation coefficient of 0.62 (95%CI 0.30; -0.81; p-value<0.001). Notably, the state of Rio Grande do Sul, despite not having the highest HDI (0.771), had the highest number of procedures per 1 million inhabitants (266.8) (Figure 2). The per capita income also showed a positive correlation with this comparison. A Spearman correlation coefficient of 0.48 (95%CI 0.11; -0.73; p-value=0.010) was observed in relation to surgeries. In the HDI analysis, the state of Rio Grande do Sul stood out, as it had more than double (266.8 versus 129.5) the number of procedures performed per 1 million inhabitants compared to the state with the highest per capita income, São Paulo (Figure 3).

## Discussion

Lung resection is widely recognized as the gold standard for therapeutic treatment of lung cancer in early stages (5). This procedure is indicated mainly for non-small cell carcinomas, which affect 85.0% of lung cancer cases, representing the most prevalent form of the disease (6). This surgical approach offers greater long-term survival when compared to other therapeutic modalities in patients with stage I and II non-small cell carcinomas. Exploring regional inequalities without access to this treatment becomes essential, since such disparities can limit the benefits provided by advances in early detection and innovative surgical techniques.

This study revealed significant correlations between socioeconomic indicators and health outcomes for lung cancer patients in Brazil. These results demonstrated the positive association between the HDI and the *per capita* income of the states with the number of surgical procedures performed, corroborating previous findings on the influence of the socioeconomic context on accessibility to treatment (7-9). A significant upward trend was identified in the number of procedures performed to treat bronchial and lung neoplasms, indicating the growing burden of lung cancer and the urgent need for improvements in health infrastructure.

Analysis of variance shows statistically significant differences (p-value<0.001) between the macro-regions of Brazil in the number of procedures performed, indicating distinct patterns in the use of health services. Geographic location and unequal distribution of highly complex health services help explain regional disparities in access to health care (7-10). The meta-analysis carried out in 2013 (11) highlighted the role of socioeconomic conditions in the allocation of resources and accessibility to health services. It has been suggested that patients with lower socioeconomic status had less access to specialist lung cancer treatments, corroborating this study and highlighting the global trend of health inequality driven by economic and geographic factors (11). The Southeast and South of Brazil are home to the country’s main university centers and well-equipped infrastructure. These regions tend to have more robust and higher quality hospital infrastructure, as well as specialized services, facilitating the population’s access to more complex procedures (12). The North region faces logistical challenges and a lack of distribution of health services, with a significant part of the population living in remote areas, far from the main health centers (13,14). 

The novelty of this study lies in identifying specific disparities in the performance of surgical procedures, highlighting how states with similar socioeconomic indices can present substantial differences in the provision of these services. The state of Rio Grande do Sul, for example, despite not having the highest HDI in the country, leads in the number of procedures per 1 million inhabitants (266.8). This data contradicts the expected trend and suggests that other factors, such as local health policies, can mitigate traditional socioeconomic limitations, pointing to new directions for future research. 

There was a significant increase of 51.6% in the number of procedures for the treatment of bronchial and pulmonary neoplasms between 2014 and 2023. This growth reflected advances in early detection methods and screening policies, which enabled faster diagnoses and a greater number of surgical interventions (15). Implementation of minimally invasive techniques, such as thoracoscopic lobectomy, has been associated with faster recovery and lower complication rates. This constitutes an important strategy for minimization of costs by reducing the length of hospital stay, benefiting the public health system and the patients (16-19). These results corroborate the literature that highlights how technological innovations and screening initiatives have promoted increased access to effective cancer treatments (15,20). 

Another relevant aspect of this research was the analysis of gender disparities in the surgical context. Although men had a higher incidence of lung cancer diagnoses (54.7%), women underwent a slightly higher number of surgical procedures (50.6% vs. 49.4%). This data complements the literature, which points to differences in behavior regarding the search for health care and better prognoses among women (8.21). The tendency for diagnosis at earlier stages and the lower prevalence of comorbidities in women may partly explain this pattern, but these results suggest that these differences should be explored in more detail in future investigations. 

This study had limitations that should be considered when interpreting the results. The database of the Department of Information and Informatics of the Unified Health System was used, a source of secondary data that may vary in accuracy and completeness between different regions. This variability may affect the comparability of results and may not fully capture regional disparities in access to and use of health services (22). This research did not consider individual patient factors, such as comorbidities, lifestyle behaviors, and genetic predispositions, which may influence both lung cancer outcomes and access to care. 

The ecological design of the study also limited the ability to establish causal relationships between socioeconomic factors and health outcomes. As the data collected were retrospective, there was a lack of detailed information on factors that may influence the trend, in addition to changes in surgical indication criteria or resource availability. Therefore, measurement bias cannot be ruled out and is a potential limitation of this study. Although associations between socioeconomic indicators and health service use have been observed, potential confounding factors, such as cultural differences in tobacco use, variations in awareness of lung cancer symptoms, and disparities in care-seeking behaviors across regions, may further affect access to care and treatment outcomes. 

This study investigated the influence of socioeconomic factors on the surgical treatment of lung cancer in Brazil and revealed significant disparities between the country’s regions. The South had the highest number of procedures per 1 million inhabitants. The North and Northeast presented lower numbers, reflecting inequalities in the distribution of health services in the country. A positive correlation was observed between HDI and per capita income with the number of procedures, highlighting the importance of socioeconomic development in diagnosis and treatment. Analysis by genders showed a higher incidence of diagnoses among men, but women underwent more surgical procedures, suggesting differences in health-seeking behaviors and disease progression. 

Technological advances and public policies have also contributed to the considerable increase in procedures over the years, although this has not been uniform across all regions. The data limitations from the Department of Information and Informatics of the Unified Health System, including the lack of detail and underreporting, highlighted the need for improvements in the quality of health records. This study highlighted the scarcity of strategies focused on contributing to more equitable access to surgical treatment of lung cancer in Brazil, especially in regions with greater socioeconomic disparities, with investments in health infrastructure and policies that reduce inequalities.

## Data Availability

The database used in the research is available at: https://datasus.saude.gov.br/ (23)
