# Supplementary material for: Epidemiological profile of surgical treatment of lung cancer: retrospective analysis, Brazil, 2014-2023
Source: Epidemiol Serv Saude. 2025 Aug 4;34:e20240427. doi: 10.1590/S2237-96222025v34e20240427.en (PMC12334236; doi:10.1590/S2237-96222025v34e20240427.en)
Supplement: Translation

# Translation: Perfil epidemiológico do tratamento cirúrgico do câncer de pulmão: análise retrospectiva, Brasil, 2014-2023

**DOI:** 10.1590/S2237-96222025v34e20240427.pt

**Authors:** Arthur Minas Alberti, Lucas Kieling, Pedro Bortoluzzi Escobar da Silva, Fernando Silvestre Azambuja, Luísa Godoy, Franco Piccolotto Concolatto, Gabriele Eckerdt Lech, Airton Tetelbom Stein

**Affiliations:** 1 Universidade Federal de Ciências da Saúde de Porto Alegre, Faculdade de Medicina, Porto Alegre, RS, Brasil; 2 Pontíficia Universidade Católica do Rio Grande do Sul, Faculdade de Medicina, Porto Alegre, RS, Brasil

**Keywords:** Neoplasias Pulmonares, Procedimentos Cirúrgicos Operatórios, Desigualdades de Saúde, Fatores Socioeconômicos, Estudos Retrospectivos

## Abstract

**Objetivo:**

Analisar as influências socioeconômicas no tratamento cirúrgico do câncer de pulmão no Brasil e fornecer reflexões para propor estratégias mais eficazes no enfrentamento dessa doença.

**Métodos:**

Dados de 2014 a 2023 foram extraídos do Sistema de Informações Hospitalares do Sistema Único de Saúde brasileiro e do Instituto Brasileiro de Geografia e Estatística. Analisaram-se o sexo e a região de procedência dos pacientes que realizaram procedimentos cirúrgicos de câncer de pulmão. As análises estatísticas foram realizadas utilizando os softwares GraphPadPrism 8.0.1 e R (versão 4.4.2)

**Resultados:**

Analisaram-se 20.805 procedimentos cirúrgicos e 112.118 diagnósticos de neoplasias malignas dos brônquios e pulmões no Brasil entre 2014 e 2023. As mulheres representaram 50,6% dos procedimentos. Os homens responderam por 54,7% dos diagnósticos. Não houve diferença significativa entre os sexos na realização de procedimentos ou consultas (p-valor>0,05). A região Sul apresentou a maior taxa de procedimentos (20,1/1 milhão de habitantes/ano). A região Norte teve a menor (3,5/1 milhão). Identificou-se aumento de 51,6% nos procedimentos ao longo da década, com correlações positivas entre o índice de desenvolvimento humano (r=0,62; p-valor<0,001) e a renda per capita (r=0,48; p-valor 0,010) com procedimentos.

**Conclusão:**

Fatores socioeconômicos parecem influenciar o acesso ao tratamento cirúrgico do câncer de pulmão no Brasil, com disparidades regionais significativas, especialmente nas regiões Norte e Nordeste. O aumento nos procedimentos reflete avanços tecnológicos e políticas públicas, mas desigualdades no acesso persistem. Investimentos em infraestrutura e políticas equitativas são essenciais para enfrentar essas desigualdades.

**Aspectos éticos**

Esta pesquisa utilizou bancos de dados de domínio público e anonimizados.:

## Introdução

A incidência e a mortalidade do câncer de pulmão apresentam desafios significativos de saúde pública, com implicações de grande alcance para os sistemas de saúde e as comunidades afetadas. Destacaram-se disparidades regionais na mortalidade por câncer de pulmão no Paraná, Brasil, oferecendo a visão sobre a influência de fatores socioeconômicos nesse cenário (1). A correlação entre as taxas de mortalidade e os indicadores como o índice de desenvolvimento municipal ressalta a conexão entre o status socioeconômico dos pacientes e o acesso ao tratamento (2,3). 

Há, na literatura, análises realizadas sob um escopo nacional. Pesquisadores do Rio Grande do Norte revelaram panorama semelhante (2), indicando alta prevalência de diagnósticos de câncer de pulmão em estágio avançado em todo o Brasil. Sugeriu-se a associação crítica entre fatores como envelhecimento populacional, renda per capita e disparidades regionais. A relação entre esses indicadores e a incidência da doença apontou a necessidade urgente de entender e abordar as disparidades no acesso oportuno aos serviços de saúde.

Em 2017, pesquisadores chineses (4) indicaram que a influência do contexto socioeconômico na incidência e mortalidade do câncer de pulmão perpassava as divisas do Brasil, caracterizando um fenômeno global. A crescente incidência entre mulheres em países como Brasil, Espanha e Chipre, juntamente com o aumento das taxas de mortalidade em regiões mais desenvolvidas, destacou a complexidade dos fatores que moldam esse panorama epidemiológico (4).

Este artigo teve como objetivo realizar a análise das influências socioeconômicas no tratamento cirúrgico do câncer de pulmão no Brasil. Ao examinar essa relação complexa entre fatores sociais e biológicos de risco, pretendeu-se identificar as disparidades existentes e fornecer reflexões para propor estratégias mais eficazes e equitativas no enfrentamento dessa doença.

## Metódos

### Delineamento do estudo

Trata-se de estudo observacional, transversal e retrospectivo, que analisou dados de 2014 a 2023. Este foi conduzido com base nos protocolos Strengthening the Reporting of Observational Studies in Epidemiology e Sex and Gender Equity in Research, visando aumentar a transparência do estudo e garantir a equidade de sexo.

### Contexto

Os dados foram coletados de duas bases brasileiras de dados secundários: o Sistema de Informações Hospitalares do Sistema Único de Saúde (SIH-SUS) e o Instituto Brasileiro de Geografia e Estatística (IBGE). As informações referiram-se ao número de procedimentos cirúrgicos realizados em todo o território nacional, estratificando-o em cinco macrorregiões geográficas: Norte, Nordeste, Centro-Oeste, Sudeste e Sul. O período analisado foi de 1º de janeiro de 2014 a 31 de dezembro de 2023.

### Participantes

Incluíram-se todos os pacientes submetidos a procedimentos cirúrgicos para o tratamento de câncer de pulmão financiados pelo Sistema Único de Saúde (SUS), registrados no SIH-SUS, com diagnósticos classificados sob a Classificação Estatística Internacional de Doenças e Problemas Relacionados com a Saúde (CID-10) C34 (neoplasia maligna dos brônquios e pulmões). Não foram aplicados critérios de exclusão, assegurando a inclusão de todas as internações registradas no período de estudo.

### Variáveis

As variáveis analisadas incluíram aspectos demográficos, como sexo, faixa etária e etnia; características clínicas, como o número de diagnósticos e procedimentos cirúrgicos realizados; e fatores socioeconômicos, como o índice de desenvolvimento humano (IDH) e a renda per capita por macrorregião geográfica.

### Fontes de dados/mensuração

Os dados clínicos e demográficos foram obtidos do SIH-SUS, enquanto os dados socioeconômicos foram extraídos do Censo Demográfico 2022 do IBGE. As informações foram padronizadas para análise, com o índice de procedimentos ajustado para 1 milhão de habitantes por estado e região. 

### Viés

Esforços foram realizados para minimizar vieses, incluindo o uso de fontes oficiais e amplamente validadas, como o SIH-SUS e o IBGE, e a realização de ajustes populacionais para comparar as taxas de procedimentos entre estados e regiões. Foi realizada avaliação da normalidade dos dados anteriormente às análises estatísticas, garantindo a validade dos testes aplicados.

### Tamanho do estudo

O tamanho da amostra foi determinado pela totalidade dos dados disponíveis no período de estudo. Foram analisados 20.805 procedimentos cirúrgicos realizados e 112.118 diagnósticos de neoplasias malignas dos brônquios e pulmões registrados entre 2014 e 2023.

### Métodos estatísticos

Análises descritivas de acordo com sexo e macrorregião foram reportadas com frequências e porcentagens. Para variáveis categóricas, foram realizados: testes de Shapiro-Wilk para verificar a normalidade dos dados; teste t não pareado para comparar os sexos; modelo de Prais-Winsten para analisar tendências anuais no número de diagnósticos e procedimentos; teste de análise de variância unidirecional com teste de post-hoc de Tukey para avaliar diferenças regionais; e correlação de Spearman para identificar associações entre IDH, renda per capita e número de procedimentos. As análises estatísticas foram realizadas utilizando os softwares GraphPad Prism 8.0.1 e R (versão 4.4.2).

## Resultados

Entre 2014 e 2023, foram reportados 20.805 procedimentos cirúrgicos pulmonares, bem como 112.118 diagnósticos de neoplasmas malignos dos brônquios e pulmões, de acordo com o CID-10 C34. Do número total de procedimentos cirúrgicos realizados, 10.529 (50,6%) foram em mulheres e 10.276 (49,4%) em homens. Em relação aos diagnósticos de câncer de pulmão, 61.273 (54,7%) foram em homens e 50.845 (45,4%) em mulheres.

**Tabela 1:** Comparação das diferenças médias dos índices de procedimentos ajustados com teste de Tukey, intervalos de confiança de 95% (IC95%) e p-valores ajustados. Regiões brasileiras, 2014-2023

Regiões	Diferença média	(IC95%) da diferença	p-valor
Norte versus Nordeste	-2,21	(-4,20; -0,22)	0,023
Norte vs. Centro-Oeste	-2,60	(-4,59; -0,61)	0,005
Norte vs. Sudeste	-8,29	(-10,28; -6,30)	<0,001
Norte vs. Sul	-16,56	(-18,55; -14,57)	<0,001
Nordeste vs. Centro-Oeste	-0,39	(-2,38; 1,60)	0,981
Nordeste vs. Sudeste	-6,08	(-8,07; -4,09)	<0,001
Nordeste vs. Sul	-14,35	(-16,34; -12,36)	<0,001
Centro-Oeste vs. Sudeste	-5,69	(-7,68; -3,70)	<0,001
Centro-Oeste vs. Sul	-13,96	(-15,95; -11,97)	<0,001
Sudeste vs. Sul	-8,27	(-10,26; -6,28)	<0,001

O teste t não pareado comparando procedimentos entre homens e mulheres não foi estatisticamente significativo (p-valor 0,693). O teste foi bilateral com estatística t de 0,400 e 18 graus de liberdade. O número médio de procedimentos foi de 1.028 para homens e 1.053 para mulheres, com diferença de 25,30 (erro-padrão da média -63,16; intervalo de confiança de 95% [IC95%] -107,4; -158,0; p-valor 0,693; r^2^=0,009). O teste F comparando variâncias resultou na estatística F de 3,522 com 9 e 9 graus de liberdade e p-valor 0,074, indicando nenhuma diferença significativa nas variâncias. Para consultas, o teste t não pareado resultou na diferença também não significativa (p-valor 0,064). Este foi um teste bilateral com estatística t de 1,970 e 18 graus de liberdade. A diferença média foi -1.043 (erro-padrão da média 529,4; IC95% -2,16; 69,46; r^2^=0,177), sugerindo o tamanho de efeito moderado. O teste F comparando as variâncias resultou na estatística F de 1,604 com 9 e 9 graus de liberdade e p-valor 0,492, indicando nenhuma diferença significativa nas variâncias. O tamanho da amostra para ambas as colunas A e B foi 10. 

Ao comparar os indicadores de procedimentos de cada região, o Sul ocupou o primeiro lugar com 20,1 procedimentos cirúrgicos para ressecção de neoplasias pulmonares e brônquicas por 1 milhão de habitantes por ano. Seguiram-se o Sudeste com 11,8, o Centro-Oeste com 6,1, o Nordeste com 5,7 e o Norte com 3,5 cirurgias para tratamento do câncer de pulmão por 1 milhão de habitantes por ano (Figura 1A).

**Figura 1:** Índice de procedimentos cirúrgicos nas regiões do Brasil (A) e número de procedimentos realizados no país de 2014 a 2023 (B)

A tendência de aumento estatisticamente significativa foi observada no número de procedimentos realizados para tratar neoplasmas brônquicos e pulmonares, após a correção da autocorrelação serial pelo modelo de Prais-Winsten (p-valor<0,001) (Figura 1B). Com a aplicação desta análise, observou-se que a tendência de crescimento do número de procedimentos cirúrgicos ao longo dos anos se manteve significativa (coeficiente=81.03; IC95% 43.53; -118.53; p-valor<0,001). O teste de Durbin-Watson indicou a redução da autocorrelação serial (Durbin-Watson=1.695; p-valor=0,045). A diferença foi 51,6%, com 1.608 procedimentos em 2014 e 2.438 em 2023 (r^2^=0,876). O teste de análise de variância revelou diferença significativa entre as médias do índice de procedimentos das cinco regiões (F (4,45) = 182,2, p-valor<0,001). O valor R^2^ foi 0,942, indicando bom ajuste do modelo, o que sugeriu diferenças significativas entre as médias das regiões. 

Correlações positivas podem ser observadas entre o IDH de um estado e o número de procedimentos cirúrgicos, com o coeficiente de correlação de Spearman de 0,62 (IC95% 0,30; -0,81; p-valor<0,001). Notavelmente, o Rio Grande do Sul, apesar de não ter o maior IDH (0,771), possuiu o maior número de procedimentos por 1 milhão de habitantes (266,8) (Figura 2). A renda per capita também mostrou correlação positiva com essa comparação. Foi observado o coeficiente de correlação de Spearman de 0,48 (IC95% 0,11; -0,73; p-valor 0,010) em relação às cirurgias. Na análise do IDH, o Rio Grande do Sul se destacou, pois tinha mais do que o dobro (266,8 versus 129,5) do número de procedimentos realizados por 1 milhão de habitantes comparado ao estado com a maior renda per capita, São Paulo (Figura 3).

**Figura 2:** Correlação entre índice de desenvolvimento humano (IDH) dos estados e seu índice de procedimentos cirúrgicos. 2014-2023

**Figura 3:** Correlação entre renda per capita em reais (R$) dos estados brasileiros e seu índice de procedimentos cirúrgicos. 2014-2023

## Discussão

A ressecção pulmonar é amplamente reconhecida como o padrão-ouro para o tratamento terapêutico do câncer de pulmão nos projetos iniciais (5). Esse procedimento é indicado principalmente para carcinomas de não pequenas células, que atingem 85,0% dos casos de câncer de pulmão, representando a forma mais prevalente da doença (6). Essa abordagem cirúrgica oferece maior sobrevida a longo prazo quando comparada a outras modalidades terapêuticas em pacientes com carcinomas de não pequenas células nos estádios I e II. Explorar as desigualdades regionais sem acesso a este tratamento torna-se fundamental, uma vez que tais disparidades podem limitar os benefícios proporcionados por avanços na detecção precoce e técnicas cirúrgicas inovadoras.

Este estudo revelou correlações significativas entre indicadores socioeconômicos e desfechos de saúde para pacientes com câncer de pulmão no Brasil. Estes resultados demonstraram a associação positiva entre o IDH e a renda per capita dos estados com o número de procedimentos cirúrgicos realizados, corroborando achados anteriores sobre a influência do contexto socioeconômico na acessibilidade ao tratamento (7–9). Identificou-se tendência significativa de aumento no número de procedimentos realizados para tratar neoplasias brônquicas e pulmonares, indicando o crescente ônus do câncer de pulmão e a necessidade urgente de melhoria na infraestrutura de saúde.

A análise de variância mostra diferenças estatisticamente significativas (p-valor<0,001) entre as macrorregiões do Brasil no número de procedimentos realizados, apontando padrões distintos no uso dos serviços de saúde. A localização geográfica e a distribuição desigual de serviços de saúde de alta complexidade ajudam a explicar as disparidades regionais no acesso à saúde (7-10). A meta-análise realizada em 2013 (11) destacou o papel das condições socioeconômicas na alocação de recursos e na acessibilidade aos serviços de saúde. Foi sugerido que pacientes com menor posição socioeconômica tinham menor acesso a tratamentos especializados para o câncer de pulmão, corroborando este estudo e enfatizando a tendência global de desigualdade em saúde impulsionada por fatores econômicos e geográficos (11). O Sudeste e o Sul brasileiros abrigam os principais centros universitários do país e infraestrutura bem equipada. Essas regiões tendem a ter infraestrutura hospitalar mais robusta e de maior qualidade, além de serviços especializados, facilitando o acesso da população a procedimentos mais complexos (12). A região Norte enfrenta desafios logísticos e falta de distribuição dos serviços de saúde, com boa parte significativa da população residindo em áreas remotas, distantes dos principais centros de saúde (13,14). 

O ineditismo deste estudo reside em identificar disparidades específicas na realização de procedimentos cirúrgicos, evidenciando como estados com índices socioeconômicos semelhantes podem apresentar diferenças substanciais na oferta desses serviços. O Rio Grande do Sul, por exemplo, apesar de não possuir o maior IDH do país, lidera em número de procedimentos por 1 milhão de habitantes (266,^8^). Esse dado contradiz a tendência esperada e sugere que outros fatores, como políticas locais de saúde, podem mitigar as limitações socioeconômicas tradicionais, apontando novas direções para futuras pesquisas. 

Houve aumento significativo de 51,6% no número de procedimentos para o tratamento de neoplasmas brônquicos e pulmonares entre 2014 e 2023. Esse crescimento refletiu avanços nos métodos de detecção precoce e nas políticas de rastreamento, que possibilitaram diagnósticos mais rápidos e maior número de intervenções cirúrgicas (15). A implementação de técnicas minimamente invasivas, como a lobectomia toracoscópica, tem sido associada a recuperação mais rápida e menores taxas de complicações. Isso constitui importante estratégia de minimização de custos pela diminuição do tempo de internação, beneficiando o sistema público de saúde e os pacientes (16-19). Estes resultados corroboram a literatura que destaca como as inovações tecnológicas e as iniciativas de rastreamento têm promovido aumento no acesso a tratamentos oncológicos eficazes (15,20). 

Outro aspecto relevante desta pesquisa foi a análise de disparidades de sexo no contexto cirúrgico. Embora os homens apresentassem maior incidência de diagnósticos de câncer de pulmão (54,7%), as mulheres foram submetidas ao número ligeiramente maior de procedimentos cirúrgicos (50,6% vs. 49,4%). Esse dado complementa a literatura, que aponta diferenças de comportamento em relação à busca por cuidados de saúde e melhores prognósticos entre as mulheres (8,21). A tendência de diagnóstico em estágios mais iniciais e a menor prevalência de comorbidades em mulheres podem explicar, em parte, esse padrão, mas estes resultados sugerem que essas diferenças devem ser exploradas mais detalhadamente em investigações futuras. 

Este estudo apresentou limitações que devem ser consideradas ao interpretar os resultados. Foi utilizado o banco de dados do Departamento de Informação e Informática do Sistema Único de Saúde, fonte de dados secundários que podem variar em precisão e completude entre diferentes regiões. Essa variabilidade pode afetar a comparabilidade dos resultados e talvez não capture totalmente as disparidades regionais no acesso e uso dos serviços de saúde (22). Esta pesquisa não considerou fatores individuais dos pacientes, como comorbidades, comportamentos de estilo de vida e predisposições genéticas, que podem influenciar tanto os desfechos do câncer de pulmão quanto o acesso aos cuidados. 

O desenho ecológico do estudo também limitou a capacidade de estabelecer relações causais entre fatores socioeconômicos e desfechos de saúde. Como os dados coletados foram retrospectivos, houve ausência de informações detalhadas sobre fatores que podem influenciar a tendência, além de mudanças nos critérios de indicação cirúrgica ou na disponibilidade de recursos. Portanto, viés de aferição não pode ser descartado e caracteriza como potencial limitação deste estudo. Embora tenham sido observadas associações entre indicadores socioeconômicos e uso dos serviços de saúde, possíveis fatores de confusão, como diferenças culturais no uso do tabaco, variações na conscientização sobre os sintomas do câncer de pulmão e disparidades nos comportamentos de busca por atendimento entre as regiões, podem afetar ainda mais o acesso aos cuidados e os resultados do tratamento. 

Este estudo investigou a influência dos fatores socioeconômicos no tratamento cirúrgico do câncer de pulmão no Brasil e revelou disparidades significativas entre as regiões do país. O Sul apresentou o maior número de procedimentos por 1 milhão de habitantes. O Norte e o Nordeste apresentaram números menores, refletindo desigualdades na distribuição dos serviços de saúde no país. Observou-se a correlação positiva entre o IDH e a renda per capita com o número de procedimentos, destacando a importância do desenvolvimento socioeconômico no diagnóstico e tratamento. A análise por sexo mostrou maior incidência de diagnósticos entre os homens, mas as mulheres se submeteram a mais procedimentos cirúrgicos, sugerindo diferenças nos comportamentos de busca de saúde e na progressão da doença. 

Os avanços tecnológicos e as políticas públicas também contribuíram para o aumento considerável nos procedimentos ao longo dos anos, embora isso não tenha sido uniforme em todas as regiões. As limitações dos dados do Departamento de Informação e Informática do Sistema Único de Saúde, incluindo a falta de detalhes e subnotificação, apontaram a necessidade de melhorias na qualidade dos registros de saúde. Este estudo enfatizou a escassez de estratégias focadas em contribuir para o acesso mais equitativo ao tratamento cirúrgico do câncer de pulmão no Brasil, especialmente em regiões com maiores disparidades socioeconômicas, com investimentos em infraestrutura de saúde e políticas que reduzam as desigualdades.

## Data Availability

O banco de dados utilizado na pesquisa está disponível em: https://datasus.saude.gov.br/(23)
